# Open Reduction and Stabilization Without Internal Fixation for the Treatment of Displaced Radial Neck Fractures

**DOI:** 10.7759/cureus.99573

**Published:** 2025-12-18

**Authors:** Shu Yoshizawa, Hideaki Ishii, Misato Sakamoto, Kayo Yamada, Takahiro Maeda, Tomoyasu Homma, Osahiko Tsuji, Hiroyasu Ikegami

**Affiliations:** 1 Department of Orthopedic Surgery, Toho University Ohashi Medical Center, Tokyo, JPN

**Keywords:** artificial bone, children, posterior interosseous nerve palsy, radial head open reduction, radial neck fracture, reduction

## Abstract

Background

Radial neck fractures are a rare injury in both adults and children. In adults, dislocated fractures of the radial neck are commonly treated using open reduction and internal fixation with headless compression screws or plate osteosynthesis. In children, isolated fractures are most commonly treated using percutaneous fixation. However, these methods are associated with the risk of posterior interosseous nerve palsy and the need for wire removal. Thus, firm fixation without the use of internal fixation is preferable. In this study, we aimed to evaluate open reduction and stabilization of displaced radial neck fractures without internal fixation.

Methods

Eight patients who underwent surgery for radial neck fractures were recruited from the institutional registry. Using a chisel, the fracture line was gently reduced from the fracture site where the radial head was maximally displaced. If a bone defect occurred and instability was observed, the defect was filled with an artificial bone graft substitute. Reduction was maintained with cast immobilization for two weeks. The angulation and translation of the fracture line was assessed.

Results

The median age of the study participants was 18.4 years. The mean follow-up period was 33.8 months. The distribution of Judet types was as follows: type II in three cases, type III in four cases, and type IV in one case. Artificial bone was used in six cases and not used in two cases. The mean preoperative tilt angle was 33.4°, and the mean preoperative lateral shift angle was 5.6 mm. Immediately after surgery, the anatomical position was successfully maintained in all patients. The mean bone union period was 62.1-80.3 days. The mean Quick Disabilities of the Arm, Shoulder, and Hand (QuickDASH) score was 1.1. The final range of motion (ROM) achieved was 152° of flexion, +4° of extension, 86° of supination, and 84° of pronation. The mean pain levels at rest and during motion, as assessed using the visual analog scale (VAS), were 0.6. No cases of malalignment, avascular necrosis, nerve palsy, premature physeal closure, or cubitus valgus were observed.

Conclusions

Position maintenance is achieved without internal fixation in both children and adults. Good fixation without internal fixation depends on the intraoperative stability. If instability is observed, artificial bone filling should be the first choice, and if instability persists, the use of an internal fixative should be considered without hesitation.

In this study, we demonstrated that open reduction and stabilization of displaced radial neck fractures without internal fixation yields satisfactory clinical and radiological outcomes.

## Introduction

Radial neck fractures are a rare injury in both adults and children [1]. In adults, comminuted and dislocated fractures of the radial neck are commonly treated using open reduction and internal fixation with headless compression screws or a plate [2-4]. In children, isolated fractures account for 5-10% of all elbow injuries [5,6] and are most commonly treated using percutaneous fixation using either the intrafocal pinning or the Metaizeau method. However, posterior interosseous nerve palsy is primarily associated with intrafocal pinning, whereas wire removal applies to the Metaizeau method, to avoid potential misinterpretation. Thus, firm fixation without the use of internal fixation is preferable. Therefore, in this study, we aimed to evaluate open reduction and stabilization of displaced radial neck fractures without internal fixation.

## Materials and methods

Eight patients who underwent surgery for radial neck fractures, between April 2020 and October 2023, were recruited from the institutional registry. All patients or parents of patients provided informed consent to this study. This retrospective study was approved by the Toho University Ohashi Medical Center Ethics Committee (approval number: H23121). None of the patients underwent closed reduction before surgery. The operation was performed using Kocher's lateral approach.

**Figure 1:**
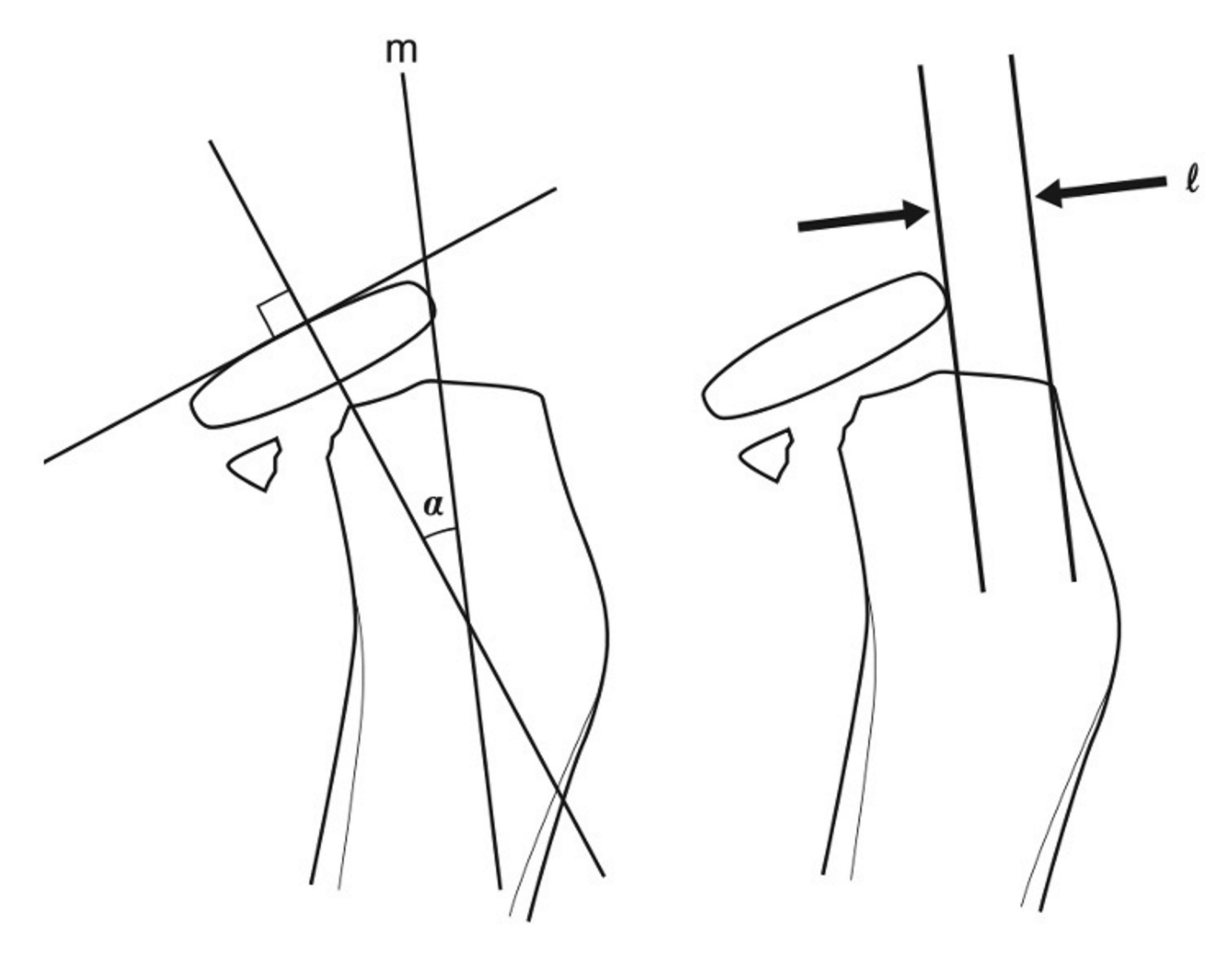
Measurement of angulation and translation of radial neck fractures α: tilt angle; m: neck axis; l: lateral shift Image Credit: Authors (modified from [7])

Interposed capsular or ligamentous structures that blocked the reduction were cut. Using a chisel, the fracture line was gently reduced from the fracture site where the radial head was maximally displaced. If a bone defect occurred and instability was observed, the defect was filled with a block-type artificial bone graft substitute. Finally, fracture reduction was evaluated using dynamic fluoroscopy. Full supination and pronation were achieved without instability, confirmed using intraoperative visualization and fluoroscopy. Subsequently, the annular ligament and capsule were carefully sutured. Reduction was maintained with cast immobilization at 90° of elbow flexion and a mid-forearm position for two weeks. After the two-week immobilization period, active and passive flexion-extension exercises of the elbow joint were initiated, followed by instruction in active and passive pronation-supination exercises beginning two weeks later. The angulation and translation of the fracture line was assessed, as shown in Figure 1 [7]. The angle between the radial neck axis and the perpendicular to the articular surface of the radial head epiphyseal angle was measured. The degree of transverse displacement was defined as the distance between the most displaced bone fragment and the lateral aspect of the radial shaft.

The fractures were classified according to the Judet classification system, and clinical outcomes were assessed using the Quick Disabilities of the Arm, Shoulder, and Hand (Quick DASH) score [8], range of motion (ROM) in extension/flexion of the elbow, pronation and supination, bone union period, pain at rest, and motion according to a visual analog scale (VAS). DASH, VAS, and ROM were assessed at the time of the final follow-up evaluation. Radiographic examination confirmed radial neck alignment, the presence of avascular necrosis, cubitus valgus, and premature epiphyseal closure. At the final follow-up, standard anteroposterior and lateral radiographs of both elbows were obtained.

## Results

Baseline characteristics of the patients and results are shown in Table 1. The median age of the study participants (five male and three female) was 18.4 years (range, 8-44 years). Among the patients, fractures occurred in both the right and the left arms (n=4 each). The mean follow-up period was 33.8 months (range, 13-96 months). The distribution of Judet types was as follows: type II in three cases, type III in four cases, and type IV in one case.

**Table 1:** Baseline characteristics of the patients and results. QuickDASH: Quick Disabilities of the Arm, Shoulder, and Hand; ROM: range of motion; VAS: visual analog scale

Variable	
Sex	
Female	3
Male	5
Age	18.4 years (8-44)
Fracture site	
Right arm	4
Left arm	4
Judet types	
Type Ⅱ	3
Type Ⅲ	4
Type Ⅳ	1
Mean follow-up period	33.8 months (range, 13-96)
Mean preoperative tilt angle	33.4° (range, 25-62°)
Mean preoperative lateral shift	5.6 mm (range, 4.5-7.3)
Mean bone union period	65.1±14.3 days (range, 46-95)
Mean QuickDASH	1.1 (range, 0-4.5)
Final ROM	152° of flexion, +4° of extension, 86° of supination, and 84° of pronation
Final VAS	0.6

Artificial bone was used in six cases and not used in two cases. The mean preoperative tilt angle was 33.4° (range, 25-62°), and the mean preoperative lateral shift angle was 5.6 mm (range, 4.5-7.3 mm). Immediately after surgery, the anatomical position was successfully maintained in all patients. The mean bone union period was 65.1±14.3 days (range, 46-95 days), with no cases of malunion. The mean QuickDASH score was 1.1 (range, 0-4.5). The final ROM achieved was 152° of flexion, +4° of extension, 86° of supination, and 84° of pronation. The mean pain levels at rest and during motion, as assessed using VAS, were 0.6. No cases of malalignment, avascular necrosis, nerve palsy, premature physeal closure, or cubitus valgus were observed.

**Figure 2:**
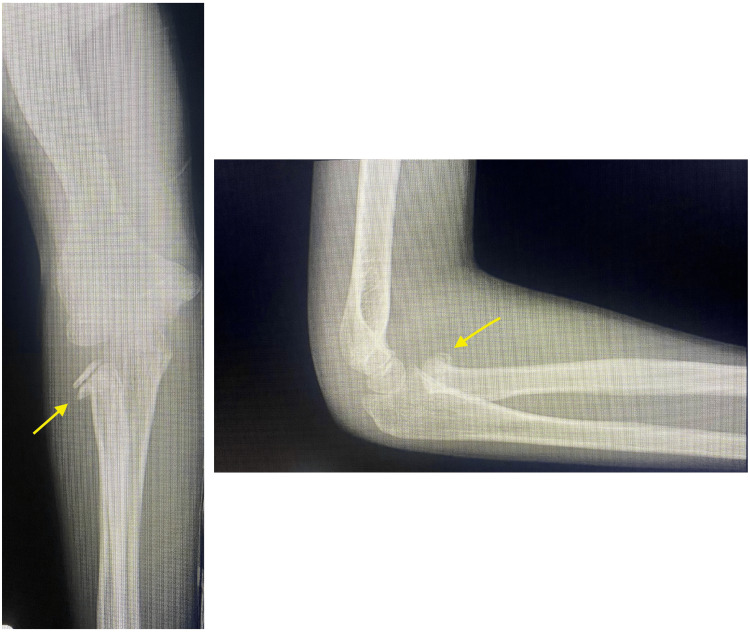
A simple radiograph revealed a fracture of the right radial neck (Judet type II).

**Figure 3:**
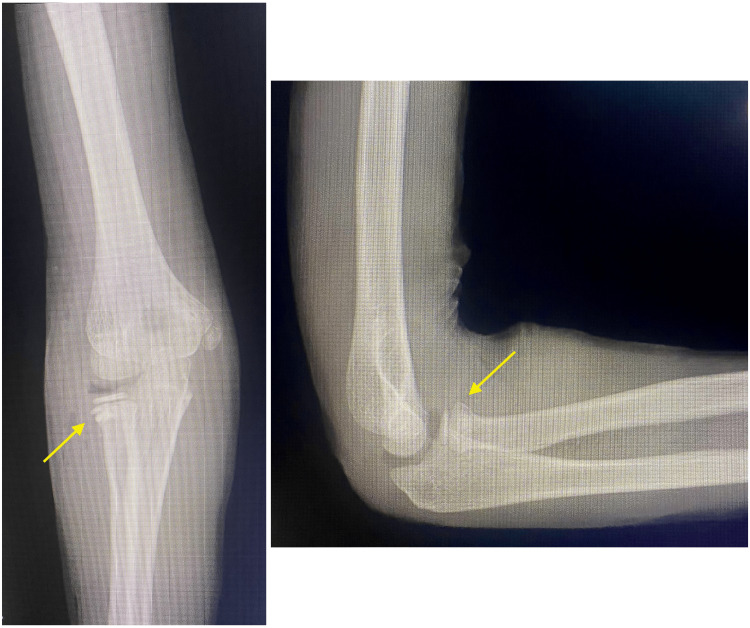
After the fracture was reduced, a bone defect was observed, and the fracture was filled with an artificial bone substitute.

**Figure 4:**
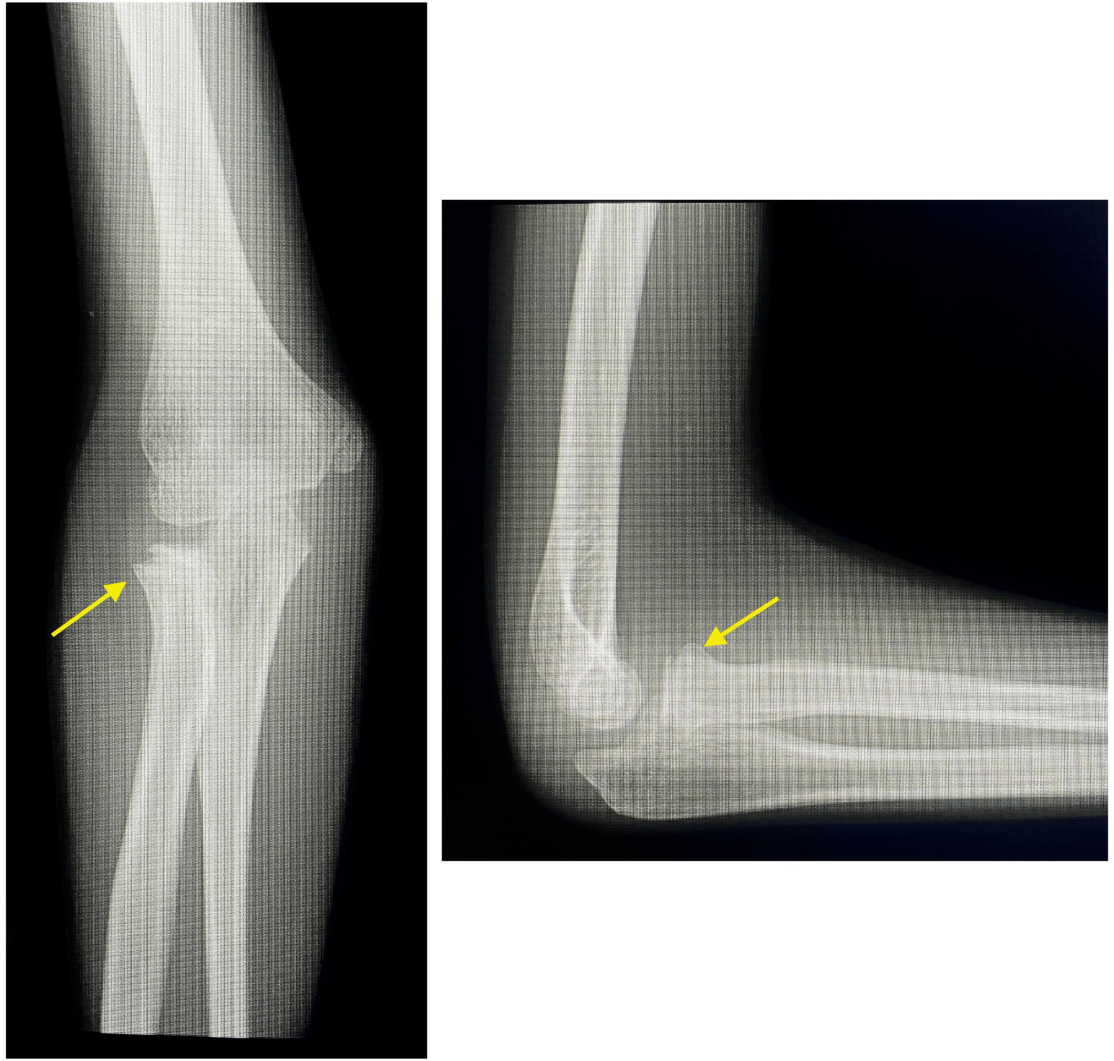
One year after surgery, simple radiographs showed no dislocation and bony fusion.

We also present a representative case involving a 10-year-old boy who was injured during a fall. A simple radiograph revealed a fracture of the right radial neck (Judet type II) (Figure 2). Surgery was performed seven days after the injury. After the fracture was reduced, a bone defect was observed, and the fracture was filled with an artificial bone substitute (Figure 3). The cast immobilized the elbow joint at 90° of flexion and the forearm in a neutral position for two weeks. One year after surgery, no limitation was found in the ROM, and simple radiographs showed no dislocation or bony fusion (Figure 4).

## Discussion

Treatment options for radial neck fractures are determined by the degree of displacement, angular deformity, and patient's age. Most non-displaced or minimally displaced radial neck fractures can be treated with closed reduction and casting, leading to good outcomes [9,10]. Fractures with a higher degree of displacement typically require surgical intervention. However, surgical intervention is associated with severe complications, including radioulnar synostosis, osteonecrosis, rotational impairment, posterior interosseous nerve palsy, the need for wire removal, and premature physeal closure, especially after more invasive procedures. Therefore, appropriate follow-up is necessary.

Furthermore, in children, radial head and neck fractures are challenging to treat. Limited treatment data has been published, and reports on optimal treatment strategies remain controversial [11,12]. Loss of motion is the most common cause of poor outcomes [13]. 

There are many kinds of surgical treatment such as closed reduction without fixation, closed reduction with percutaneous fixation using either the intrafocal pinning or Metaizeau method, open reduction, or a mix of the options. However, there is controversy on the fracture angulation criteria for surgical treatment.

Reduction of radial neck fractures can be difficult and may result in re-displacement. In 48 fractures series treated with closed reduction without internal fixation, 36 fractures had mild to severe malalignment [14]. And closed reduction without internal fixation results in a limited ROM in more than 60% of cases in fractures with an angular deformity of more than 30°. Therefore, a recent review recommended percutaneous fixation of closed reduction [15]. However, manipulation with a Kirschner wire near the epiphyseal line may cause epiphyseal abnormalities and the risk of posterior interosseous nerve palsy [16].

Therefore, instead of this approach, we used open reduction and stabilization of displaced radial neck fractures without internal fixation. If a bone defect occurred and instability was observed after open reduction, the defect was filled with an artificial bone graft substitute. The use of artificial bone grafts eliminates wire-associated complications (iatrogenic nerve injury such as posterior interosseous nerve palsy) and the need for wire removal.

In this study, stability was achieved, in all Judet types, using the surgical method without internal fixation, regardless of the degree of dislocation. The anatomic position was maintained without postoperative corrective loss, although postoperative casting was performed for only two weeks, and excellent results were achieved in all cases. The study included a wide range of participants (children, adolescents, and adults). Therefore, we also demonstrated that, without internal fixation, position maintenance is achieved in both children and adults.

The radial head is held in place by the radial notch of the ulna and annular ligament, which encircles approximately 4/5 of the circumference of the radial head. Due to the anatomical features of the proximal radial-ulnar joint, the reduction position can be easily maintained without internal fixation. Therefore, internal fixation may not always be necessary if the patient undergoes open reduction.

Although one case of Judet type III had a good outcome with no dislocation, it is necessary to carefully consider which cases are suitable for this procedure. Good fixation without internal fixation depends on the intraoperative stability. If instability is observed, artificial bone filling should be the first choice, and if instability persists, the use of an internal fixative should be considered without hesitation.

This study has several limitations. First, this was a retrospective study, limiting the comparison of different methods and treatment strategies. Additionally, this study included a small sample size. Because of the infrequency of this particular fracture, the number of cases was small. Radiological assessment of early angular deformity depends on a precise imaging technique. Due to pain immediately after injury, some initial radiographs did not include anteroposterior or lateral views. Furthermore, inter- and intra-observer analyses were not performed for the methods used to measure initial radial head-to-shaft angle and displacement. Finally, the study includes pediatric patients (as young as eight years old); the follow-up period is insufficient to adequately assess late complications such as premature physeal closure or growth disturbances. So, longer-term follow-up is typically required to evaluate these outcomes in skeletally immature patients.

## Conclusions

In this study, we demonstrated that open reduction and stabilization of displaced radial neck fractures without internal fixation yields satisfactory clinical and radiological outcomes. This method has the advantage of eliminating various risks associated with internal fixation such as posterior interosseous nerve palsy, the need for wire removal, and limited ROM. However, this study did not have a sufficient number of cases to establish clear criteria for this method, and further investigation is warranted.
